# Development of a 3D-Printed High Temperature Resin Cecal Fistula Implant for Long-Term and Minimally Invasive Access to the Gut Microbiome

**DOI:** 10.3390/nu13124515

**Published:** 2021-12-17

**Authors:** Dulce M. Minaya, Noah L. Weinstein, Krzysztof Czaja

**Affiliations:** Department of Biomedical Sciences, College of Veterinary Medicine, University of Georgia, Athens, GA 30602, USA; dulceminaya@gmail.com (D.M.M.); Noah.Weinstein@uga.edu (N.L.W.)

**Keywords:** microbiome, microglia, brain, cecum, 3D-printed fistula, fecal transplant, dysbiosis

## Abstract

Microbiota dysbiosis has been associated with chronic diseases ranging from gastrointestinal inflammatory and metabolic conditions to neurological changes affecting the gut-brain neural axis, mental health, and general well-being. However, current animal studies using oral gavage and gnotobiotic animals do not allow for non-invasive long-term access to gut microbiome. The purpose of the present study was to evaluate the feasibility of 3D-printed fistula implants through the body wall and into the cecum of rats to obtain long-term access to gut microbiome. Cecal fistulas were designed and 3D-printed using a high temperature resin (Formlabs; acrylic and methacrylic mixture). Nine male Sprague-Dawley rats underwent the fistula implantation. Food intake, body weight, and body fat were measured to determine the impact of fistula manipulation. Gut microbiome, vagal afferents in the hindbrain, and microglia activation were analyzed to determine if fistula implantation disrupted the gut-brain neural axis. We found that the procedure induced a transient decrease in microbial diversity in the gut that resolved within a few weeks. Fistula implantation had no impact on food intake, body weight, fat mass, or microglia activation. Our study shows that 3D-printed cecal fistula implantation is an effective procedure that allows long-term and minimally invasive access to gut microbiome.

## 1. Introduction

The gut microbiome has become an area of intense study for contemporary researchers. Studies have shown that the gut microbiome plays a crucial role in early physiological development in humans, influencing everything from bone growth to the outcome of diseases such as asthma and certain neurodevelopmental disorders [1]. Germ-free animals (GF), which lack all microorganisms including the gut microbiome, exhibit altered expression of motor control and anxiety-like behavior [2], decreased microglia development in the brain, inhibited responses to spatial and temporal stimuli, and increased blood-brain barrier permeability [3]. Additionally, they have been shown to have arrested the growth of capillary networks [4]. Perhaps the most relevant association between modern western health issues and the microbiome is the link between diet induced obesity and gut dysbiosis [5,6,7]. Gut dysbiosis refers to the imbalance of bacterial species compositions, which can lead to deleterious effects on the intestinal lining and overall immune function. Crohn’s disease, ulcerative colitis (UC), and pouchitis are all the result of the pathogenic immune response following antigenic stimulation by microbiota in the gut, due to mucosal barrier defects [8]. Consumption of a high-fat/high sugar diet rapidly, within a few weeks, increased ratio of Firmicutes/Bacteroidetes species and decreased in overall microbiota diversity [5,6,7]. An increased ratio of Firmicutes/Bacteroidetes species and decreased microbial diversity are known markers of microbiota dysbiosis. In addition, it has been reported that analysis of the gut microbiome could be a tool in the diagnosis of colorectal cancer. Cordero, et al. reported that colorectal cancer patients exhibit a significantly different microbiota composition, with *Firmicutes* being the most abundant phylum, when compared to healthy subjects and patients with adenoma [9].

Further, understanding the means by which dysbiosis can occur and be repaired is also important in capitalizing on its role as a potential biomarker for the onset of serious oncological conditions such as the aforementioned colorectal cancer (CRC). Current research shows that microbes such as *Escherichia coli* and *Bacteroides fragilis*, among countless others, may have a more direct linkage to the onset and development of CRC than previously thought [10]. These microbes are thought to affect the onset of cancers such as CRC by affecting genome stability, resistance to cell death, and proliferative signaling [9]. As such, the early detection of gut dysbiosis may indicate an increased proclivity towards the onsets of CRC, or even an indication of its initial emergence. Though methods for screening for CRC continue to advance, the disease remains the number three most common cancer worldwide, and second most deadly, though the prognosis improves drastically with early detection [11]. Thus, more thoroughly understanding the means by which dysbiosis can occur and be repaired may allow researchers to more effectively use the microbiome as a biomarker in the screening of cancers such as CRC, and provide clinicians with pointed preemptive treatment through microbial intervention to reduce the likelihood of the onset, or even utilize immunotherapy improve the prognosis, of CRC through the restoration of a healthy gut microbiome.

With multiple studies showing the harmful effects of gut dysbiosis on a wide range of health endpoints, the question of how to efficiently restore gut health has moved to the forefront of gut microbiota research. One of the most prominent treatments currently being researched is the fecal transplant, wherein gut dysbiosis is treated with the introduction of “healthy” bacteria, often from the donor’s feces. Though this therapy is still limited primarily to the treatment of *Clostridioides difficile* in current clinical settings, it continues to show promise in treating many diverse diseases with links to gut dysbiosis, such as inflammatory bowel diseases [9,10]. It is likely that gut microbiome manipulation will become a prime avenue for the treatment of a wide range of diseases. 

Most studies involving microbiota transplant follow a standard experimental plan: subject animals are administered a powerful prophylactic antibiotic or are raised under GF conditions to prepare the animal for the transplant, before delivering the transplant through oral gavage, or, somewhat less commonly, with their food [11]. The use of these protocols to study the gut microbiome, however, introduces some limitations to the knowledge we can acquire about the normal physiological function of the gut microbiome [12]. 

As previously mentioned, GF animals exhibit a host of developmental and behavioral abnormalities. It has also been noted that the ceca of germ-free rats could grow to up to five times larger than that of standard animals. These same authors note that the enlarged cecum also often suffers from fluid buildup leading to thinned cecal walls [13]. Thus, using GF animals that exhibit altered brain function, cytokine expression, and physiology to study these processes as they relate to the introduction of a certain microbiota profile would cast doubt on conclusions that could be drawn from such studies. 

Due to the cost and difficulty in acquiring and maintaining germ-free laboratory animals, treating the subject animals with prophylactic antibiotics has become a popular method for “preparing” the subject for transplant. However, the reality is that the efficacy of prophylactic antibiotics for this purpose is questionable; with outcomes including that transplant following antibiotic intake shows no uptake of donor microbiota by the recipients compared to the untreated recipients [14]. It has also been shown that microbiota transplants after antibiotic treatment can result in the overgrowth of certain antibiotic resistant strains of bacteria after recolonization of the microbiome, often hampering the ability of researchers to understand the effects of certain bacterial strains [15]. 

Transplant options following the preparation of the subject animal are similarly inefficient and rely almost entirely on oral gavage. Though select studies have detailed methods for preparing rodent chow containing donor microbiota, this methodology is labor intensive and would be a poor choice for large scale studies or those requiring specialized and standardized diets [16]. The greatest benefit to oral gavage is the precision with which the dose and timing of the liquid can be administered. However, oral gavage is a stressful procedure for rodents [17], and some gavaged animals may experience aspiration of stomach contents into the lungs [18,19]. In addition, oral gavage causes unavoidable irritation to the esophagus [20]. This damage may be as mild as inflammation and fibrosis, or as serious as perforation and ulceration [18]. 

**Figure 1:**
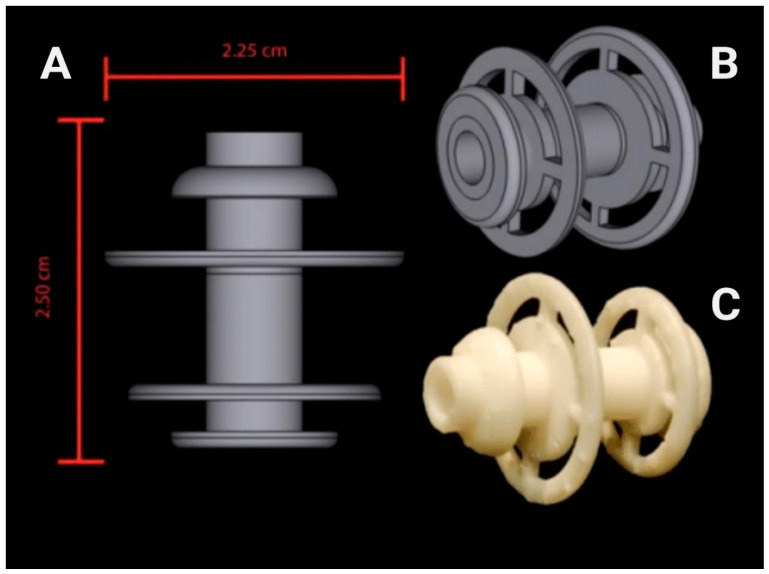
Fistula Design. Fistulas were designed in Shapr3D Software (A; lateral view; B; oblique view). Formlabs High Temp Resin-printed fistula (C).

Thus, there is a meaningful need to explore alternate methods of microbiome manipulation and long-term and minimally invasive access to the gut microbiome. However, the studies revealing the methods for direct gut microbiome access in animal models are limited and do not provide information about long-term impact of the procedure on gut dysbiosis [21,22]. Therefore, the goal of this study was to evaluate the feasibility of a 3D-printed fistula, designed in our laboratory (Figure 1), and implanted through the abdominal wall and into the cecum of rats to obtain direct access to gut microbiome without long-term dysbiosis. Results of the study show that implanting a cecal fistula did not induce long-term phenotypic dysbiosis, which was also evidenced by no effect on the integrity of vagal gut-brain connections. Moreover, cecal fistula manipulation did not impact food intake, body weight, and fat mass. Our study shows that 3D-printed cecal fistula implantation is an effective procedure that allows long-term and minimally invasive access to the gut microbiome and enables future studies on microbiome transplant by minimizing limitations of currently used methods in the field of microbiome research.

## 2. Materials and Methods

### 2.1. Animals

Male Sprague–Dawley rats (*n* = 12; ~300 g; Envigo, Indianapolis, IN, USA) were housed individually in conventional polycarbonate shoe-box cages in a temperature-controlled vivarium with ad libitum access to regular chow pellets (PicoLab rodent diet 20, product #5053, Fort Worth, TX, USA) and water. Rats were maintained on a 12:12-h light: dark cycle with lights on at 0700-h and allowed to acclimate to laboratory conditions for one week prior to starting experiments. All animal procedures were approved by the University of Georgia Institutional Animal Care and Use Committee and conformed to National Institutes of Health Guidelines for the Care and Use of Laboratory. 

### 2.2. Experimental Timeline

The experiment had a duration of 12 weeks. During the first week, baseline measures of food intake, body weight, and body composition were taken. Then, the animals underwent the fistula implantation surgery and were allowed to recover for three weeks (weeks 2–4). After recovery, the animals were randomly assigned to following groups: HFD/F (*n* = 3), Chow/C (*n* = 3), and Chow/F (*n* = 3). The HFD/F group was switched to a high-fat/high sugar diet. The other two groups remained on regular chow. During the last four weeks of the experiment, the HFD/F and Chow/F groups were restrained using a plastic decapicone three times per week, their fistulas were opened, and gut contents were removed using a 3 mL plastic pipette.

### 2.3. Food Intake, Body Weight, and Body Composition

Following the acclimation period, rats were maintained on regular chow for an additional three days to obtain a baseline measure of food intake, body weight, and composition. Henceforth, 24-h food intake was measured once a week. Body weight and body composition were measured weekly using a minispec LF 110 BCA Analyzer (Bruker Corp., Billerica, MA, USA). 

Following their recovery from surgery, animals were randomly divided into three experimental groups: Chow/C (consuming rodent chow and fistula not opened/manipulated = control; *n* = 3), Chow/F (consuming rodent chow and cecal content collected via fistula; *n* = 3), and HFD/F (consuming high-fat diet and cecal content collected via fistula; *n* = 3). Both the Chow/C and Chow/F groups remained on standard pellets of rat chow, and the HFD/F cohort was switched to a 45% fat, 20% sucrose diet (Research Diet #D12451, New Brunswick, NJ, USA). Rats were maintained on their respective diets for seven weeks. During the last three weeks on their respective diet, the Chow/F and HFD/F groups had their fistulas opened and closed three times a week to gauge the animals’ behavioral response to the manipulation and inspect the area.

### 2.4. Fistula Design, Production and Surgery

All fistulas were designed in Shapr3D and printed on a Formlabs Form 3, 3D printer using Formlabs High Temp Resin (Formlabs Inc., Somerville, MA, USA) (Figure 1). The fistula shape was designed through a period of research and development using rat cadavers at the beginning of this study. Utilizing the rapid production nature of 3D printing, researchers were able to analyze the shape of the cadavers’ cecum and its position in the body, prototype a rough design on an Apple IPad Pro, and quickly send samples to print. This process was repeated several times to produce the final iteration of the device seen in this experiment. Upon final printing, Formlabs High Temp Resin was determined to be the most appropriate printing material for the study. Current limitations in resin printing technology mean that researchers are forced to decide between different resins with different properties, such as temperature resistance or hardness. Ultimately, it was decided that the assurance of minimal warping following the presurgical autoclave of all devices was of the utmost importance, making Formlabs High Temp Resin the clear choice for this experiment. The 10–24 thread Size, 1” long titanium hex head screw (# 94081A103; https://www.mcmaster.com/94081A103/; accessed on 12 November 2001) was used to close the fistula opening.

**Figure 2:**
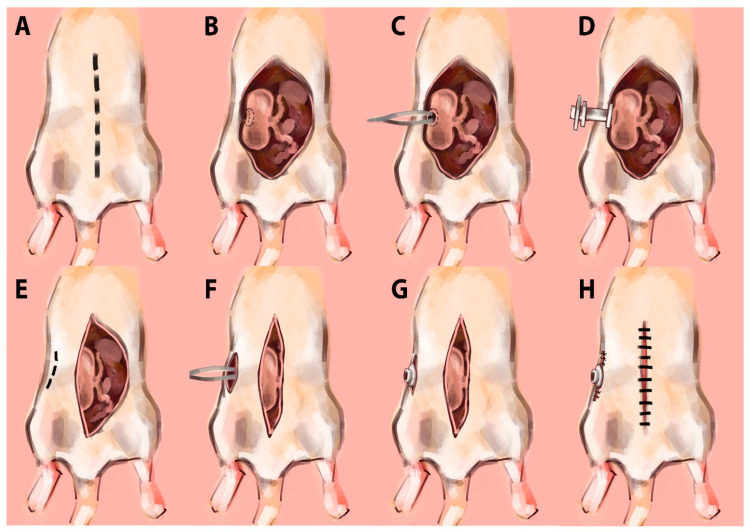
(**A**–**H**) Illustration of the surgical implantation method for the cecal fistula.

Following the acclimation period, the animals underwent fistula implantation surgery (Figure 2). On surgery day, following an overnight fast, anesthesia (3–4% isoflurane and oxygen) was administered to the rats in a small rodent anesthesia chamber. A four-five cm midline incision was made, starting just caudal to the sternum (Figure 2A). The cecum was then be located, drawn up through the incision, and exposed. Grasping the apex of the cecum with a pair of forceps, a 12 mm diameter purse suture was placed on the outer membrane of the cecum around the incision (Figure 2B). A small 7.5 mm hole was then cut on the apex with a pair of scissors, facing the right flank of the animal where the fistula would exit, and was then spread open with a small pair of forceps (Figure 2C). The smallest, inner flange of the fistula was then placed into the incision, and the purse suture was tightened around the fistula (Figure 2D). The second flange was then secured to the serosal membrane of the cecum with four to five simple sutures around the circumference of the flange.

A second, 15 mm vertical incision was made 20 mm to the right of the first abdominal incision and stretched with a pair of forceps to accommodate the fistula (Figure 2E,F). The forceps were then inserted into the second incision, the cecum was returned to the abdomen and the external end of the fistula was pulled through the second incision with the forceps (Figure 2G). The third and outermost flange was maneuvered to rest on top of the skin, and the second incision was closed with three or four simple sutures, taking care to suture as close to the flange as possible for a tight fit (Figure 2H). The first incision was then closed with simple sutures along both the outer muscular layer and the skin. After the closure of the incisions, the rats were monitored until they emerged from anesthesia, regained righting reflex, and could move normally.

**Figure 3:**
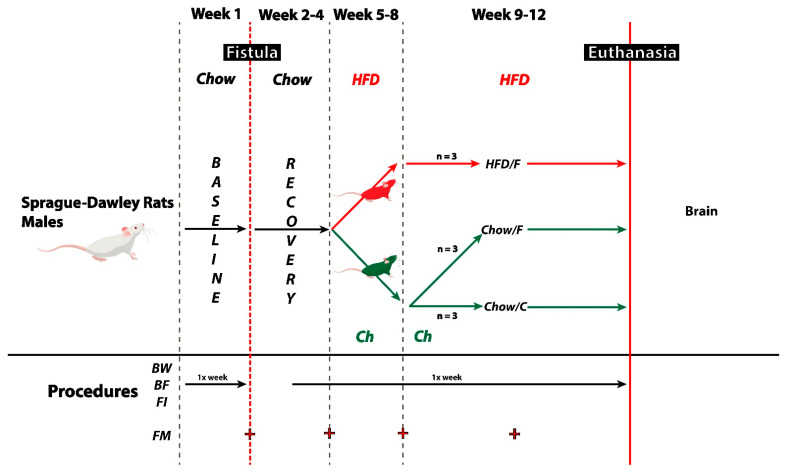
Experimental timeline. Following a ten-day acclimation period, male Sprague-Dawley rats (*n* = 9) underwent the fistula implantation surgery. After three weeks of recovery, animals were randomly assigned to following groups: HFD/F (*n* = 3), Chow/C (*n* = 3), and Chow/F (*n* = 3). The HFD/F group was switched to a high-fat/high sugar diet. The other two groups remained on regular chow. During the last four weeks of the experiment, the HFD/F and Chow/F groups were restrained using a plastic decapicone three times per week, their fistulas were opened, and gut contents were removed using a 3 mL plastic pipette. Throughout the experiment, body weight, body fat mass, and 24-h food intake measurements were recorded once a week.

During the postoperative period, the rats were monitored daily for the progression of their recovery. They received injections of buprenorphine (0.05–0.1 mg/kg IM) every 12 h to minimize post-surgery pain and gentamicin (0.1 mg/kg IM) to prevent infections. Animals were closely monitored and considered as fully recovered when body weight returned to pre-surgery body weight. Three of the 12 rats in this initial proof-of-concept trial did not have a full recovery and were removed from the experiment. See Figure 3 for an overview of the experimental timeline.

### 2.5. Microbiome Analysis

Fecal samples were collected at baseline (A), after recovery from surgery (B), four weeks after the introduction of the high energy density diet (C), and on the last day of the experiment (D). Bacterial DNA was extracted from feces using a commercial kit (Quick-DNA Fecal/Soil Microbe Miniprep Kit, cat #D6010, Zymo Research, Irvine, CA, USA). High throughput sequencing was performed using Illumina MiSeq paired-end runs (GGBC, Athens, GA, USA). Amplification targeted the V3-V4 region of the 16S ribosomal RNA genes using the following primers: S-D-Bact-041-b-S-17 (5′-CCTACGGGNGGCWGCAG-3′) forward and S-D-Bact-0785-a-A-21 (5′-GACTACHVGGGTATCTAATCC-3′) [17]. Sequences were subsequently trimmed, joined, and quality filtered. To identify Operational Taxonomic Units (OTUs) and to evaluate beta and alpha diversities, we used the Quantitative Insights Into Microbial Ecology (QIIME) software package [20]. 

### 2.6. Euthanasia

Rats were anesthetized with CO_2_ and transcranial perfused with 0.1 M phosphate-buffered saline (PBS; pH 7.4) followed by 4% paraformaldehyde. Hindbrains were harvested, post-fixed in 4% paraformaldehyde for 2-h, and immersed in 30% sucrose, 0.1% NaN_3_ (Sigma-Aldrich, St. Louis, MO, USA; pH 7.4) in PBS and stored at 4 °C until processing. Hindbrain samples were cryosectioned (Leica CM1950, Leica Biosystems, Wetzlar, Germany) at 20 μm thickness.

### 2.7. Microglia Activation and Fiber Density

Standard immunofluorescence was used to determine microglia activation and vagal afferent density in the hindbrain. Sections were incubated overnight with a primary antibody against ionized calcium-binding adaptor molecule 1 (Iba-1, Wako, Richmond, VA, USA; Cat#019-19741, RRDI: AB_839504) followed by Alexa-488 secondary antibody to visualize microglia activation as previously described [5]. In addition, hindbrain sections were incubated with GSL I-isolectin B4 biotin-conjugated (IB4, Vector Laboratories Cat#B-1205, RRDI: AB_2314661) overnight followed by ExtrAvidin-CY3 (Sigma-Aldrich, St. Louis, MO, USA; Cat#E-4142) for 2 h to visualize primary unmyelinated vagal afferents innervating the GI tract as previously described [8]. Sections were mounted in ProLong (Molecular Probes, OR) and examined under a Nikon 80-I fluorescent microscope. The area fraction of Iba1 was analyzed using Nikon Elements AR software as previously described [18,23].

### 2.8. Statistical Analysis

GraphPad Prism 7 (GraphPad Software, Inc., San Diego, CA, USA) was used to conduct statistical analyses. Data are expressed as mean ± SEM and were analyzed using ANOVA followed by Holm-Sidak multiple comparisons test. The alpha value for statistical significance was set at 0.05.

## 3. Results

### 3.1. 3D-Printed High Temp Resin Cecal Fistula Allowed Multiple Long-Term and Minimally Invasive Access to the Gut Microbiome

Multiple collections of the cecal content via 3D-printed High Temp Resin cecal fistula over a period of four weeks (Figure 3; week 9–12) proved to be an effective procedure that allowed long-term and minimally invasive access to the gut microbiome. The implantation allowed the use of a single wrap sterile transfer pipette (# HS206373C; Heathrow Scientific) to collect ~3 mL of cecal content, while inducing little to no stress to the animal during manipulation. The animals were acclimated to being restrained using plastic decapicones and displayed normal calm restraint behaviors while opening/closing the fistulas by the titanium screw.

### 3.2. A Cecal Fistula Manipulation Did Not Impact Food Intake, Body Weight, Fat Mass

**Figure 4:**
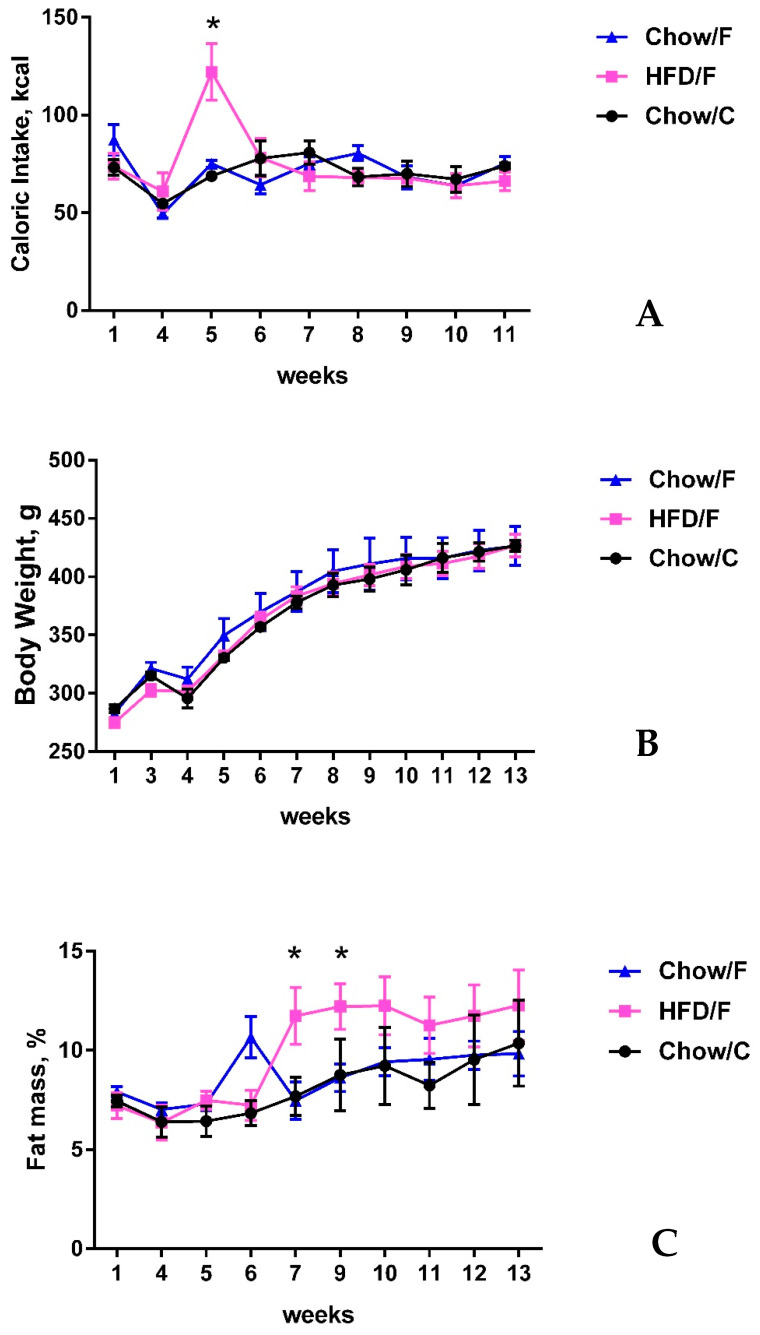
High-fat diet consumption increased body fat mass. Shown are mean + SD kcal consumed (**A**), body weight (**B**), and body fat mass (**C**) for rats fed a high-fat diet (HFD/F, *n* = 3) and rats fed regular chow (Chow/C and Chow/F, *n* = 3 respectively). 24-h food intake slightly decreased after fistula surgery but returned to pre-surgery level of intake within a few weeks. Animals switched to a high-fat diet significantly increased their caloric intake upon introduction of the diet, but caloric intake declined after one week and matched the intake of chow-fed animals for the duration of the study (**A**). There was no significant difference in body weight between the groups (**B**), but the high-fat fed cohort exhibited a significant increase in fat mass accumulation (**C**). * indicate statistical significance from week 1, * *p* < 0.05.

Group means for caloric intake, body weight, and body fat mass are shown in Figure 4A–C. There was no difference in food intake between the groups, except for a spike when the high-fat diet was first introduced to the HFD/F group (Figure 4A). This transient increase in food intake has been previously reported [5]. There was no difference in body weight (Figure 4B). Body fat mass increased significantly (*p* < 0.05) in the high-fat fed animals (Figure 4C).

### 3.3. Consumption of a High-Fat Diet, but Not Implanting a Cecal Fistula, Induced Changes in the Gut Microbiota

**Figure 5:**
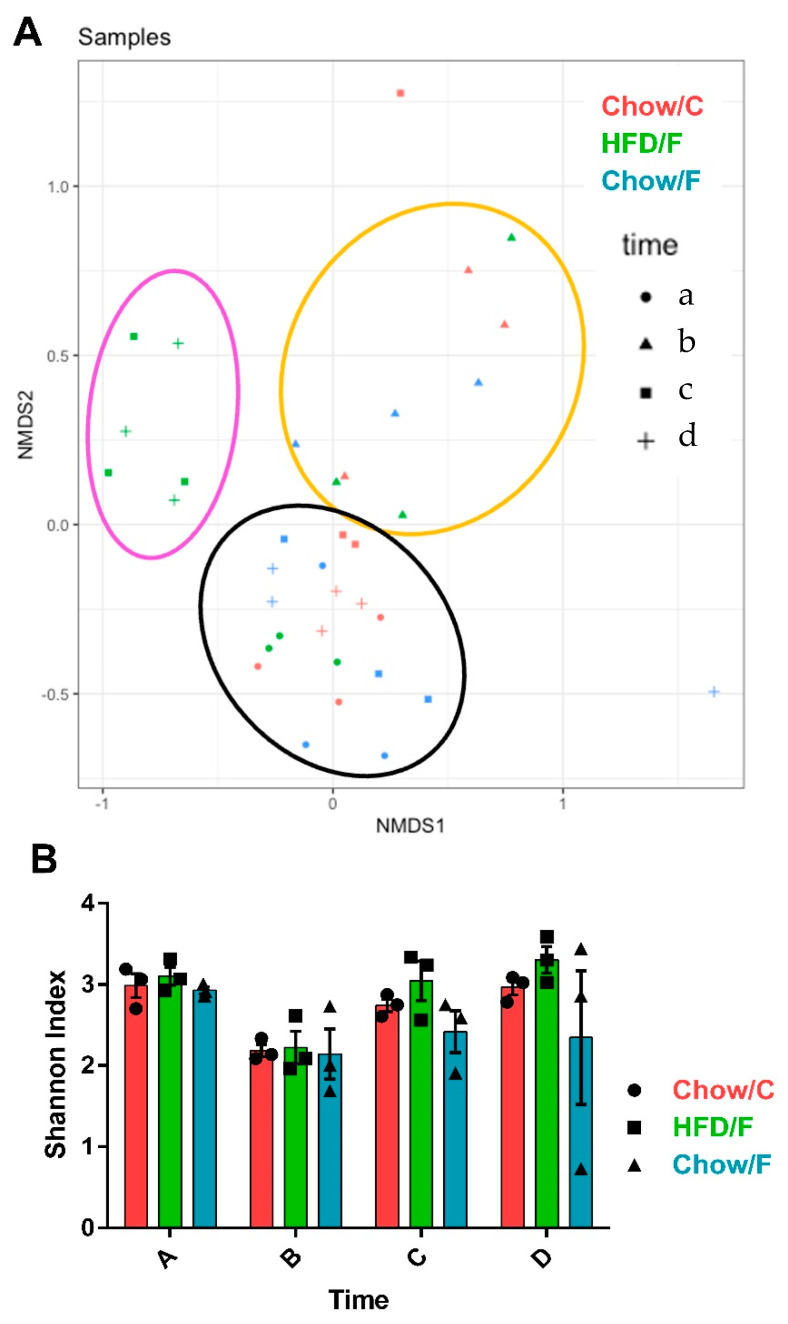
Consumption of a high-fat diet triggered remodeling of the gut microbiota. Samples were taken for microbiota analysis at four points throughout the experiment: a—before surgery, b—after recovery from surgery, c—four weeks after introduction of a high-fat diet, and d—at the end of the experiment. (**A**) Principal component analysis showed that all animals clustered together at baseline. After surgery, all animals clustered together and away from their baseline profile (b). Four weeks after introduction of the high-fat diet, the high-fat fed group clustered away from their post-surgery and baseline profile. The chow fed animals clustered with their baseline profile (c). At the endpoint, all chow fed animals clustered with their baseline profile, and the high-fat fed cohort clustered away (d). (**B**) Shannon index shown as mean + SEM for each group and time point. Bacterial diversity was slightly decreased after the surgery but recovered to pre-surgery levels within a few weeks.

Results from microbiome analysis are shown in Figure 5. Microbiome analysis showed no persisting disruption by the cecal implantation. Principal Component Analysis (PCA; Figure 5A) showed that at baseline a, all animals clustered together. After recovery from surgery, the animals clustered away from their baseline profile. Four and eight weeks after recovery from surgery, all chow-fed animals clustered together close to their baseline profile. Four and eight weeks after the introduction of the high-fat diet, high-fat animals clustered together and away from their baseline profile. Analysis of the Shannon index, a measure of species diversity, revealed a transient non-significant decrease in bacterial diversity immediately after surgery (Figure 5B). 

### 3.4. Implanting a Cecal Fistula Did Not Affect the Density of Vagal Afferents and Microglia Activation

**Figure 6:**
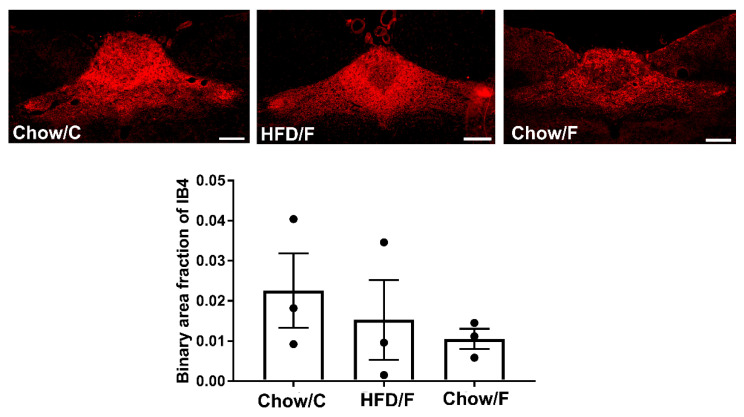
Manipulating the gut microbiome through a cecal fistula did not affect the density of vagal afferents in the intermediate NTS. Representative sections of intermediate NTS of animals fed a high-fat diet for seven weeks (HFD/F, *n* = 3) and rats fed regular chow (Chow/C and Chow/F, *n* = 3 respectively) are shown. Binary analysis of the area fraction of IB4-labelled vagal afferents revealed no significant differences in the density of labelled afferent terminals between the three cohorts. Graphs represent mean + SEM. Scale bar = 200 μm.

**Figure 7:**
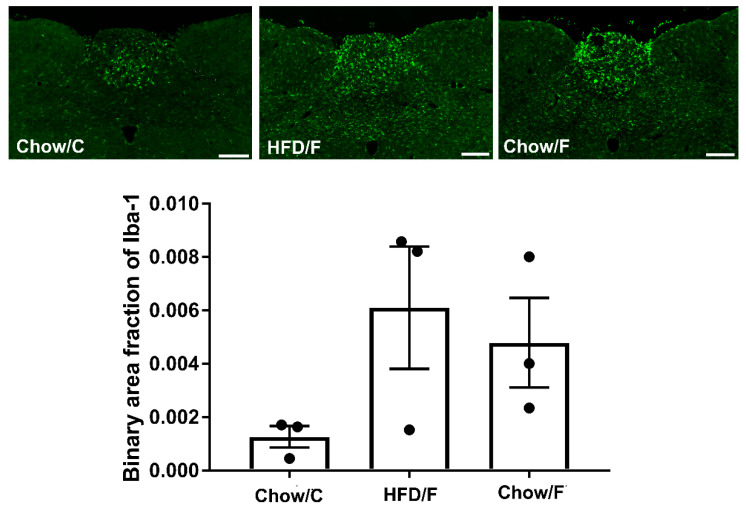
Manipulating the gut microbiome through a cecal fistula did not trigger increased microglia activation in the intermediate NTS. Representative sections of intermediate NTS of animals fed a high-fat diet for seven weeks (HFD/F, *n* = 3) and rats fed regular chow (Chow/C and Chow/F, *n* = 3 respectively) are shown. Binary analysis of the area fraction of Iba1 immunoreactivity showed no significant difference in microglia activation between the groups. We can appreciate greater individual variability in the HFD/F and Chow/F groups than in the Chow/C cohort. Graphs represent mean + SEM Iba1 intensity. Scale bar = 200 μm.

Analysis of the binary area fraction of IB4-labeled afferents in the intermediate nucleus tractus solitarius (NTS) showed no significant difference in the density of the vagal afferents among the groups (Figure 6). Similarly, immunostaining against Iba1 revealed that implanting a cecal fistula did not trigger microglia activation in the intermediate NTS (Figure 7).

## 4. Discussion

The goal of this study was to evaluate the feasibility of a 3D-printed fistula designed in our laboratory as a method for obtaining direct access to gut microbiome without inducing long-term dysbiosis following the implantation of the fistula in the cecum of rats. Results of the study show that implanting a cecal fistula did not induce long-term dysbiosis and did not affect the integrity of vagal gut-brain connections. Moreover, cecal fistula manipulation did not impact food intake, body weight, or fat mass. These results indicate the implantation of a cecal fistula is an effective procedure that allows long-term and minimally invasive access to the gut microbiome to better facilitate future studies on microbiome transplants by minimizing the limitations of currently used methods in the field of microbiome research. The surgical implantation procedure proved to be well tolerated among study animals with no alteration in their mobility or physical activity, indicating the long-term efficacy of the device. By the endpoint of the experiment, all animals were found to be in good health. 

Fistula manipulation during the cecal content (gut microbiome) sampling also did not induce changes in food intake, body weight, accumulation of fat mass, or rat behavior. Previous studies revealed that oral gavage, the most common method to manipulate gut microbiome, can induce significant stress to laboratory animals [17,24] and consequently affect these food intakes and body weight parameters [23]. Oral gavage is also prone to inducing aspiration in the subject animals. Upon retraction of the gavage cannula or needle from the rodent following delivery of the liquid, it is unavoidable for some of the dosage and stomach contents to be pulled up with the tool [18]. Studies have shown this residue to settle in the trachea and along the walls of the esophagus, or even make its way into the lungs or nasal passage of the animal resulting in aspiration pneumonia [19]. All of these negative and confounding effects of oral gavage on microbiome studies can be circumvented by cecal fistula implantation, though further comparative studies are necessary to remove other potential confounders not considered by this initial study. Moreover, as mentioned previously, the use of germ free or gnotobiotic animals in the study of the gut microbiome has several limitations due to the different morphology of the gastrointestinal organs, which affects growth of the animals [25,26]. The increased food intake, fat mass accumulation, and changes in the gut microbiome observed in the high-fat fed animals with fistula implants were similar to previously described studies and most likely are not associated with the surgical procedure [5]. 

The most noteworthy finding of the present study was that implanting a cecal fistula did not induce long-term dysbiosis. While the majority of currently reported experiments involving microbiota transplant use animals prepared for the transplant by administration of powerful prophylactic antibiotics, the use of prophylactic antibiotics is a significant limitation in the current fecal transplant methodology [14]. Due to the cost and difficulty in acquiring and maintaining germ-free lab animals, treating the subject animals with prophylactic antibiotics has become a popular method for “preparing” the subject for transplant. However, the reality is that the efficacy of prophylactic antibiotics for this purpose is questionable at best, with one study even showing that a microbiota transplant following the administration of antibiotics showed no uptick in donor microbiota compared to the untreated recipients [14]. Also highlighted by these same authors is one of the most significant consequences of antibiotic use in microbiota transplants; the overgrowth of certain antibiotic resistant strains of bacteria after recolonization of the microbiome or the damage done to the physiology of the subject animal itself [15]. Furthermore, relying on a full course of antibiotics to prepare a subject animal for microbiota transplant limits the number of transplants possible, as it would be inefficient and potentially harmful to repeatedly clean out the gut microbiome each time the donor sample is to be administered. Moreover, previously discussed stress related to oral gavage, a method used for gut microbiome transplant, could significantly alter the gut microbiome [27]. While it does take a few weeks after the implantation procedure for the gut microbiome to return to pre-surgery status, this transient alteration of gut microbiome was most likely an effect of Gentamicin intramuscular injection to prevent post-surgical infections and did not affect the integrity of vagal gut-brain connections. The minimal surgical use of antibiotics in the preparation of the rodents likely reduced the chance of inducing long-term dysbiosis supporting our methodology over traditional means of microbiome research.

And finally, the results of our study revealed that cecal fistula implantation numerically increased microglia activation and decreased the density of vagal afferents in the hindbrains of Chow/F and HFD/F rats, but the observed changes did not reach statistical significance. An increase in microglia activation and decrease of vagal projections to the hindbrain observed in the HFD/F group (rats fed a high-fat diet) was similar to previously reported observations [5,28]. Therefore, the observed changes in the HFD/F group from our study are most likely due to the diet, rather than fistula implantation. The changes in the Chow/F group need to be further investigated to confirm that fistula implantation is not affecting the integrity of vagal gut-brain connections.

While the results of the study showed improvements over currently used methods of gut microbiome manipulations, this pilot investigation also identified several potential improvements in the fistula design. Future iterations will be printed with a shortened middle section as well as a reduced diameter for the inner flanges. While the current design is functional but not fully optimized for the direct access of cecal content, it is believed that these improvements will greatly reduce the likelihood of any post-surgery complications as the rodents adapt to the implantation of the device. The 3D-printed nature of the fistula means that such improvements can be swiftly implemented and produced for future studies at little cost. Additionally, though the aforementioned high temperature resin provided an adequate research tool, advancements in 3D printing technology may make the production of titanium devices financially viable in the near future, increasing the reusability and, thus, the efficacy of the device as a research tool. 

The results of our study show that the implantation of a 3D-printed cecal fistula, using a high temperature resin, is an effective procedure that allows long-term and minimally invasive access to the gut microbiome. They expand the knowledge on previously published cecal fistulation methods [21,22] by providing data on the long-term integrity of vagal gut-brain connection and microbiome changes. Concerns about the extent to which anaerobic conditions will be maintained in the cecum after insertion of the fistula raised in previous studies [22], were indirectly addressed by our results. The oxygen entering the gut during fistula manipulation didn’t alter the fecal microbiome indicating that the intestinal flora didn’t change and didn’t introduce a different pattern of fermentation. 

While our results advance the knowledge on cecum fistulation and enable low-stress and long-term access to gut microbiome, there are two major limitations in this study that will be addressed in future research. First, using more animals in each group could introduce the statistical significance in microglia activation changes observed after the fistula implantation, suggesting that the procedure is altering vagal gut-brain signaling. And second, the microbiome analysis from cecal content samples, collected during the study, could directly address the influence of oxygen entering the cecum on intestinal flora and fermentation. Therefore, we plan to address the above limitations in future studies. 

## 5. Conclusions

In conclusion, our results demonstrate that 3D-printed cecal fistula implantation is an effective procedure that allows long-term and minimally invasive access to the gut microbiome in rats without the confounding negative side effects observed with other methods currently in use. Moreover, the manipulation of gut microbiome via cecal fistula could enable potential treatments for a wide array of diseases. Studies are now needed to explore this method further, in order to better understand the mechanisms by which researchers can influence the development or treatment of disease through the manipulation of the gut microbiome.

## Data Availability

The data supporting reported results can be obtained from the corresponding author.
